# Engagement With Text Messaging Improves Cardiovascular Medication Adherence: Secondary Analysis of a Randomized Controlled Trial

**DOI:** 10.2196/80794

**Published:** 2025-11-24

**Authors:** Rowan Shore-Plavec, Rachel Zucker, Thomas J Glorioso, Sheana Bull, Larry A Allen, Joseph J Saseen, Katy E Trinkley, Pamela Peterson, Joy Waughtal, P Michael Ho

**Affiliations:** 1 College of Natural Sciences and Mathematics, University of Denver, Denver, CO, United States; 2 Institute for Health Research, Kaiser Permanente Colorado, 16601 East Centretech Parkway, Aurora, CO, 80011, United States, 1 303-739-3595; 3 Clinical Assessment, Reporting, and Tracking Program, Office of Quality and Patient Safety, Veterans Health Administration, Washington, DC, United States; 4 Clinic Chat LLC, Denver, CO, United States; 5 Division of Cardiology, School of Medicine, University of Colorado Anschutz Medical Campus, Aurora, CO, United States; 6 Department of Family Medicine, School of Medicine, University of Colorado Anschutz Medical Campus, Aurora, CO, United States; 7 Department of Clinical Pharmacy, Skaggs School of Pharmacy and Pharmaceutical Sciences, University of Colorado Anschutz Medical Campus, Aurora, CO, United States; 8 Adult and Child Center for Outcomes Research and Delivery Science (ACCORDS), School of Medicine, University of Colorado Anschutz Medical Campus, Aurora, CO, United States

**Keywords:** cardiovascular disease, medication adherence, texting, interactive chatbot, behavioral nudges

## Abstract

We conducted this secondary analysis to assess whether greater engagement with a text messaging intervention was associated with improved cardiovascular medication adherence at 12 months.

## Introduction

Medication nonadherence remains a significant barrier to the optimal management of chronic cardiovascular diseases, leading to increased morbidity and health care costs [1]. Digital interventions, particularly text-based nudges, are potential strategies for enhancing medication adherence [2-5]. The Nudge study (NCT 03973931) compared the effectiveness of three different text-based interventions versus usual care to improve medication adherence among patients with documented medication refill gaps (time between medication supply end and the next refill) [6]. There was no difference in adherence (assessed by proportion of days covered [PDC]) at 12 months between any of the intervention groups and usual care [6].

While the main Nudge study results were null [6], prior studies suggest patient engagement in mHealth interventions is critical to their success [2,7,8]. We conducted this secondary analysis to assess whether greater engagement with the text messaging interventions was associated with improved medication adherence at 12 months. We hypothesized that patients who replied to study texts would have higher medication adherence defined by a shorter gap length between medication refills and higher refill adherence.

## Methods

### Ethical Considerations

The study was deemed minimal risk, and a waiver of consent was obtained from the Colorado Multiple Institutional Review Board (18‐2779). A deidentified dataset was used for the current analyses. Patients were not compensated to participate in the study.

### Study Overview

Details of the main Nudge study have been previously described, including the inclusion and exclusion criteria [1]. For secondary analyses, we restricted the cohort to patients randomized to text messaging arms in the study. During the intervention period, patients receiving study texts could respond: (1) “STOP” to opt-out of the study; (2) “DONE” if medication had been refilled; (3) numeric response in reply to a chatbot question; or (4) free text response. We categorized patient engagement into four mutually exclusive groups: (1) “Opt-out Response” for “STOP;” (2) “Other Response” for any non‐standard, free text reply (“Multimedia Appendix 1); (3) “Standard Response” for standard replies like “DONE” or a numeric response; and (4) “No response” for patients who did not reply to any messages.

Differences between groups were tested using ANOVA for continuous variables and multiple degree of freedom chi-squared tests for categorical variables (Table 1). Adjusted PDC differences between responders and nonresponders were estimated using a Generalized Estimating Equation model with an identity link and independent covariance, adjusting for the treatment arm and relevant patient characteristics (Table 1). The median unadjusted gap lengths (calculated as the number of days from the end of medication supply to the subsequent refill) with interquartile ranges were calculated for initial enrollment gaps using Kaplan-Meier estimates. Unadjusted gap lengths and the 1-year PDC by response status were reported along with adjusted estimates of differences in PDC relative to those without a response. Analyses were performed using R software (version 4.4.1; R Foundation for Statistical Computing), with α=.05.

**Table 1.** Patient demographics, clinical characteristics and study assignment across response groups.

Variable	Counts of type of response				*P* value
	No (n=3686)	Opt-out (n=456)	Other (n=843)	Standard (n=1963)	
Study arm, n (%)					<.001
Generic	1395 (38)	155 (34)	233 (28)	541 (28)	
Behavioral	1184 (32)	139 (30)	251 (30)	731 (37)	
Behavioral + Chatbot	1107 (30)	162 (36)	359 (42)	691 (35)	
Health care system, n (%)					<.001
Denver Health	3083 (84)	277 (61)	668 (79)	1314 (67)	
UCHealth	290 (8)	100 (22)	53 (6)	261 (13)	
Veterans Affairs	313 (8)	79 (17)	122 (15)	388 (20)	
Demographics					
Age, mean (SD)	59.9 (12.9)	62.9 (13.2)	59.5 (11.2)	59.9 (12.7)	<.001
Female, (n) %	1732 (47)	189 (41)	413 (49)	929 (47)	.07
Race, n (%)					.01
American Indian or Alaska Native	37 (1)	5 (1)	12 (1)	18 (1)	
Asian	50 (1)	1 (1)	7 (1)	23 (1)	
Black or African American	636 (17)	47 (10)	137 (16)	305 (16)	
Native Hawaiian/Pacific Islander	7 (1)	0 (0)	0 (0)	4 (1)	
White	2522 (68)	362 (79)	579 (69)	1339 (70)	
Multiple	20 (1)	4 (1)	6 (1)	10 (1)	
Unknown	414 (11)	37 (8)	102 (12)	204 (10)	
Ethnicity, n (%)					<.001
Hispanic	1904 (51)	0150 (33)	470 (56)	891 (45)	
Non-Hispanic	1759 (48)	302 (66)	367 (43)	1051 (54)	
Unknown	23 (1)	4 (1)	6 (1)	21 (1)	
Spanish speaking, n (%)	1033 (28)	70 (15)	335 (4)	513 (26)	<.001
Marital status, n (%)					<.001
Married	1430 (38)	200 (44)	375 (44)	909 (46)	
Single	1545 (42)	156 (34)	288 (34)	647 (33)	
Divorced/Widowed	693 (19)	97 (21)	177 (21)	383 (20)	
Unknown	18 (1)	3 (1)	3 (1)	24 (1)	
Insurance, n (%)					<.001
Medicare	1446 (39)	213 (47)	274 (32)	658 (33)	
Medicaid	1141 (31)	90 (20)	212 (25)	477 (24)	
Commercial	649 (17)	75 (16)	239 (28)	471 (24)	
Veterans Affairs	9 (1)	3 (1)	1 (1)	10 (1)	
None	299 (8)	36 (8)	87 (10)	209 (11)	
Unknown	142 (4)	39 (8)	30 (4)	138 (7)	
At least 1 interactive voice response message, n (%)	273 (7)	57 (12)	17 (2)	269 (14)	<.001
Qualifying condition(s)^a^, n (%)					
Atrial fibrillation	206 (6)	41 (9)	40 (5)	127 (6)	.01
Coronary artery disease	537 (15)	80 (18)	93 (11)	272 (14)	.01
Diabetes mellitus	1883 (51)	229 (50)	438 (52)	924 (47)	.02
Hyperlipidemia	1634 (44)	246 (54)	382 (45)	951 (48)	<.001
Hypertension	2922 (79)	367 (8)	657 (78)	1541 (79)	.65
Medical history, n (%)					
Congestive heart failure	298 (8)	39 (9)	52 (6)	133 (7)	.10
Chronic kidney disease	355 (10)	39 (9)	61 (7)	141 (7)	.01
Cardiovascular disease	244 (7)	32 (7)	40 (5)	96 (5)	.02
Depression	675 (18)	88 (19)	164 (19)	399 (20)	.32
Prior myocardial infarction	185 (5)	23 (5)	34 (4)	83 (4)	.43
Prior revascularization	100 (3)	21 (5)	22 (3)	46 (2)	.07
Posttraumatic stress disorder	144 (4)	26 (6)	47 (6)	117 (6)	<.01
Substance abuse	170 (5)	21 (5)	39 (5)	76 (4)	.61
Baseline medication class, % (n)					
Active class					.04
1	917 (25)	97 (21)	192 (23)	522 (26)	
2	876 (24)	115 (25)	210 (25)	586 (26)	
3+	1893 (51)	244 (54)	441 (52)	935 (48)	
Study class					<.001
1	2467 (67)	345 (76)	588 (70)	1433 (73)	
2	770 (21)	70 (15)	167 (20)	361 (18)	
3+	449 (12)	41 (9)	88 (10)	169 (9)	

a This number exceeds 100% due to the fact that one patient can have more than one qualifying condition(s) for this study.

## Results

A total of 9501 patients were enrolled; 9269 had complete follow-up data (the CONSORT diagram has been published previously [6]). After excluding 2321 usual care patients, we analyzed 6948 individuals; 3262 (46.9%) responded to an intervention message. Among the 3262 responders, 456 (14%) opted out, 843 (25.8%) provided other responses and 1963 (60.2%) provided standard responses. Patients who engaged with the messages, particularly standard responders, differed significantly from nonresponders, with engagement more likely among those in certain health care systems or on fewer medications (Table 1).

Patients who engaged with the texts, regardless of response type, had a shorter gap length compared to patients who did not respond. In addition, patients who responded to any messages had a higher adherence at 12 months, which persisted after the adjustment for demographics and clinical characteristics (Table 2).

**Table 2.** Initial gap length and 1-year medication adherence (proportion of days covered; PDC) by patient response type.

Patient response type	Unadjusted		Adjusted		
	Initial gap length in days, median (IQR)	1-year PDC	Difference in PDC	95% CI	*P* value
Response					
No response (Ref)	16 (1-109)	55.9	—^a^	—	—
Any response	7 (1-29)	71	15.2	13.7-16.8	<.001
Sub-groups (Ref: No response)					
Opt-out	8 (1-41)	67.5	9.6	6.1-13.1	<.001
Other	7 (1-32)	70	14.5	12.2-16.7	<.001
Standard	7 (1-25)	72.1	16.6	14.9-18.4	<.001

a not applicable.

## Discussion

This secondary analysis assessed the association between engagement and medication adherence in a text messaging clinical trial. We enrolled a diverse patient population including a large proportion of Hispanic and Spanish-speaking patients as well as patients who are traditionally medically underserved (ie, receiving care at federally qualified health centers and veterans affairs centers). Overall, 47% of intervention arm patients engaged with texts. Any engagement was associated with shorter medication gap lengths, and higher medication adherence at 12 months. While our participants included diverse and traditionally underserved patients, these findings may not be generalizable to patients without access to or comfort with text messaging, who would have been ineligible for the parent trial.

Another potential limitation lies in our response classification criteria. Patients who submitted nonstandard responses were placed exclusively in the “Other Response” group, which may conflate ambiguous interactions with more meaningful engagement. Furthermore, we cannot determine causality between engagement and adherence given this post-hoc analysis.

Our findings are consistent with prior literature highlighting the importance of patient engagement in mHealth interventions to improve adherence and self-management [2-5]. Collectively, these findings suggest that patient engagement is a strong marker for, and is closely associated with, higher medication adherence.

## Supplementary material

10.2196/80794 Multimedia Appendix 1 Examples of text message responses from participants classified as "Other Response."

## Abbreviations

PDC: proportion of days covered
